# Antitumor activity of EGFR-specific CAR T cells against non-small-cell lung cancer cells in vitro and in mice

**DOI:** 10.1038/s41419-017-0238-6

**Published:** 2018-02-07

**Authors:** He Li, Yao Huang, Du-Qing Jiang, Lian-Zhen Cui, Zhou He, Chao Wang, Zhi-Wei Zhang, Hai-Li Zhu, Yong-Mei Ding, Lin-Fang Li, Qiang Li, Hua-Jun Jin, Qi-Jun Qian

**Affiliations:** 1 Departments of Respiratory and Critical Care Medicine, Changhai Hospital, The Second Military Medical University, 200438 Shanghai, China; 2 Department of Biliary Tract Surgery I, The Eastern Hepatobiliary Surgery Hospital, The Second Military Medical University, 200438 Shanghai, China; 3 Shanghai Cell Therapy Research Institute, 201805 Shanghai, China; 4 Laboratory of Gene and Viral Therapy, The Eastern Hepatobiliary Surgery Hospital, The Second Military Medical University, 200438 Shanghai, China; 5 Department of Biotherapy, The Eastern Hepatobiliary Surgery Hospital, The Second Military Medical University, 200438 Shanghai, China; 6 Department of Respiratory and Critical Care Medicine, Shanghai First People’s Hospital, Shanghai Jiaotong University, 200080 Shanghai, China

## Abstract

Effective control of non-small-cell lung cancer (NSCLC) remains clinically challenging, especially during advanced stages of the disease. This study developed an adoptive T-cell treatment through expression of a chimeric antigen receptor (CAR) to target human epidermal growth factor receptor (EGFR) in NSCLC. We optimized the non-viral *piggyBac* transposon system to engineer human T cells for the expression of EGFR-CAR, consisting of EGFR scFv, transmembrane domain, and intracellular 4-1BB-CD3ζ signaling domains. The modified CAR T cells exhibited expansion capability and anticancer efficacy in a time- and antigen-dependent manner in vitro as well as regression of EGFR-positive human lung cancer xenografts in vivo. EGFR-CAR T therapy is a promising strategy to improve the efficacy and potency of the adoptive immunotherapy in NSCLC. Moreover, EGFR-CAR T therapy could become a clinical application for NSCLC patients in the future.

## Introduction

Lung cancer is a frequently diagnosed malignancy. Indeed, in 2012 it was one of the leading causes of cancer-related death in both men and women worldwide^1,2^. Histologically, lung cancer is primarily classified into small-cell lung cancer (SCLC) and non-small-cell lung cancer (NSCLC); NSCLC is the most common subtype of lung cancer (making up to 85% of lung cancer cases)^1,2^. Despite several advances in early detection, prevention, and treatment of lung cancer during the past three decades, the 5-year overall survival of patients remains low, especially for those in advanced stages of disease^3^ when patients are often only first diagnosed thus making curable surgery ineffective. Furthermore, most patients are insensitive to chemoradiotherapy at advanced stages.

Recent novel strategies targeting therapy and immunotherapy are promising, although patients still experience tumor metastasis or emergence of treatment resistance^4^. Pleasingly, there has been some compelling evidence from studies ranging from targeted kinase inhibitor regimen to immunotherapy when randomized trials were compared with classical chemotherapy^5^. Thus immunotherapy could form the basis of lung cancer control in the future.

Indeed, much progress in cancer immunotherapy has recently taken place; chimeric antigen receptor (CAR) technology in particular has revolutionized our cancer therapeutic approach. Specifically, CAR is a synthetic receptor re-engineered to be expressed in T cells to target tumor-associated antigens (TAAs) on the surface of tumor cells, thus overcoming the body’s immunoreaction and immunologic tolerance without major histocompatibility complex restriction^6^.

CAR T-cell therapy has consistently produced remarkable antitumor activities in hematological system diseases (e.g., cell-derived malignancies) and use of CD19-redirected CAR T cells has generated a complete remission rate of up to 90% in acute lymphoblastic leukemia (ALL) patients^7–9^. However, to date, due to lack of appropriate TAAs, CAR T therapy of solid tumors remains challenging; on-target toxicity (caused by expression of the targeting antigens in non-tumor cells) is another major obstacle^10^.

Nevertheless, in this study, we aimed to develop a second-generation epidermal growth factor receptor (EGFR)-specific CAR T therapy depending on *piggyBac* transposon system against NSCLC in vitro and in nude mouse xenografts. Our hypothesis is based on NSCLC overexpression of EGFR as a TAA.

EGFR is a transmembrane glycoprotein and belongs to a member of the ERBB receptor tyrosine kinase family^11^. EGFR overexpression due to *EGFR* gene amplification and/or mutation has been observed in a wide range of human cancers (including >60% of NSCLC) associated with tumor recurrence, neoangiogenesis, and metastases^12^. The EGFR extracellular domain expressing on tumor cell surface does create an ideal tumor-specific and immunogenic epitope; thus EGFR could be an appropriate target for adoptive cellular immunotherapy and be approved following successful clinical trials in which monoclonal antibodies against EGFR or its variants were satisfactorily tolerated in patients^13^. Furthermore, the *piggyBac* transposon system is a non-viral strategy to facilitate a gene delivery for functional CAR T production^14^. This system introduces a plasmid that encodes a desired gene fragment into T cells and then inserts into the cell genome with the transiently expressed transposase enzyme to recognize inverted repeat sequences. A previous genome-wide study indicated that the *piggyBac* transposon led to stable integration of the transgene and is suitable for clinical application because of the non-preferential integration into proto-oncogenes and reduction of production cost compared with viral vectors^15^.

In this study, we aimed to provide useful preclinical data to further facilitate a phase I clinical trial for patients with advanced EGFR-positive cancers.

## Results

### Generation of EGFR CAR expressed T cells in vitro

**Fig. 1:**
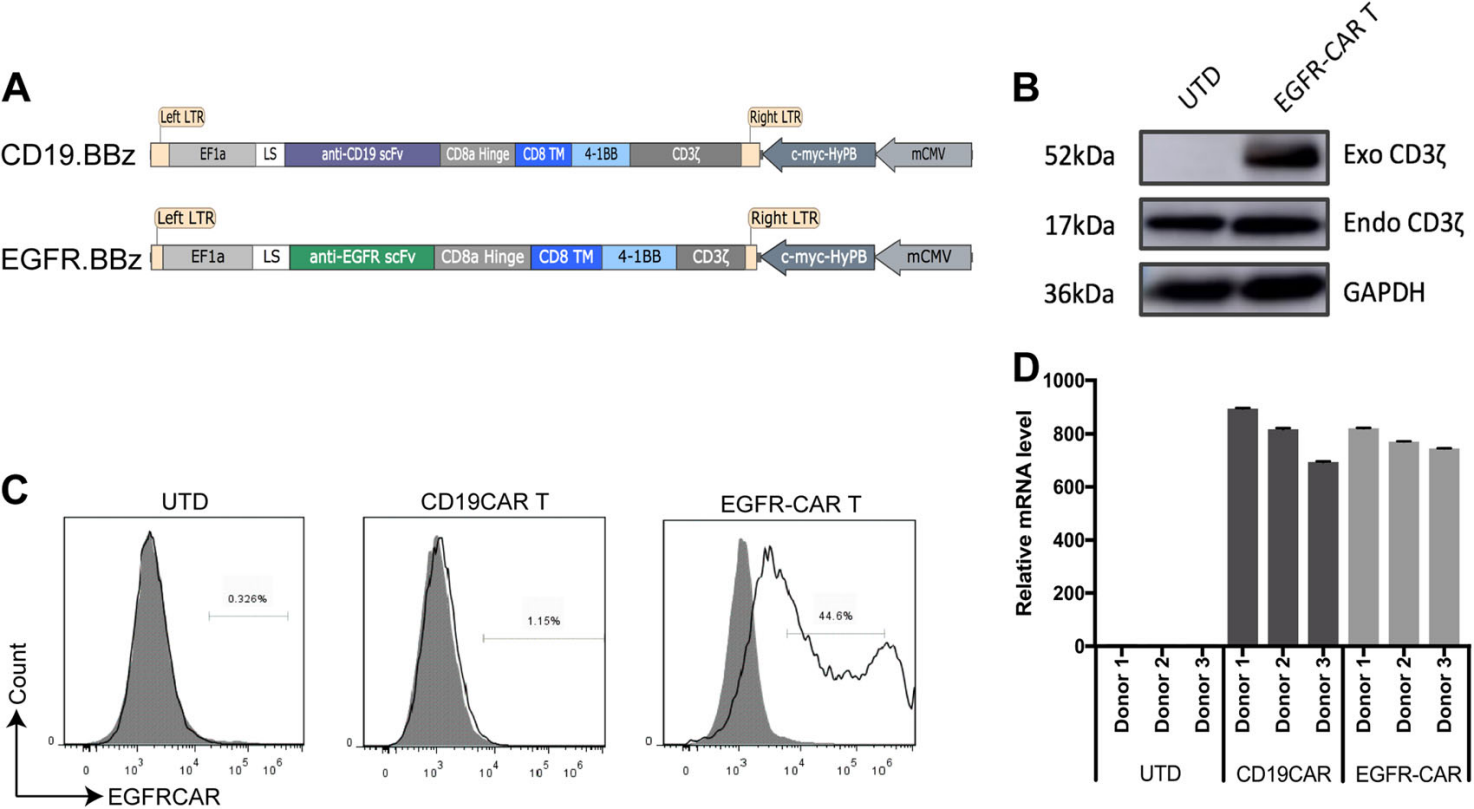
Construction and expression of CAR in EGFR-specific CAR T lymphocytes. **a** Schematic illustration of EGFR and CD19 CAR. The constructs contain EGFR or CD19 scFv, CD8α hinge and transmembrane domain, 4-1BB intracellular domain, and CD3ζ cytosolic domain. LS indicates leader signal sequence; TM transmembrane region. **b** Western blot. Expression of modified CAR protein in CAR T cells after plasmid transduction with the predicted EGFR-CAR for 52 KDa in exogenous CD3ζ, 17 KDa in endogenous CD3ζ and 36 KDa in GAPDH an endogenous control. **c** qRT-PCR. Relative expression of constructed CAR exogenous CD3ζ mRNA was detected in EGFR-CAR T cells derived from different healthy donors using RT-PCR with specific primers targeting exogenous CD3ζ. **d** Flow cytometry. Membrane-bound CAR expression in un-transduced (UTD), EGFR-CAR, or CD19 CAR T cells using flow cytometry. Histograms were gated with the percentage of positive cells

To generate EGFR CAR-expressed T cells in vitro, we first constructed plasmids carrying the CARs, which contain the anti-human single-chain variable fragment (scFv) to recognize EGFR and the *piggyBac* transposon system (Fig. 1a). The EGFR-directed CAR expression was composed of an anti-EGFR scFv fused to a CD8α hinge and transmembrane region and the intracellular signaling domains of human 4-1BB and CD3ζ motif in tandem. The CD19 CAR only containing an anti-CD19 scFv was used as a negative control for antigen-binding specificity to distinguish alloreactivity and xenoreactivity.

We then transduced these plasmids into primary human peripheral blood cells, and after efficient enrichment and expansion following approximately 10–14 days of co-culture with anti-CD3/CD28 monoclonal antibodies (mAbs) and interleukin (IL)-2, we obtained the CAR T cells, i.e., the level of the exogenous CD3ζ expression was much higher in EGFR CAR-expressed T cells than in un-transduced (UTD) T cells (Fig. 1b), while relative exogenous CD28-CD3ζ mRNA levels were also significant (Fig. 1d). The flow cytometric data showed the high T-cell transduction efficiency and expression of different proteins (Fig. 1c).

### Characterization of EGFR-CAR T cells

**Fig. 2:**
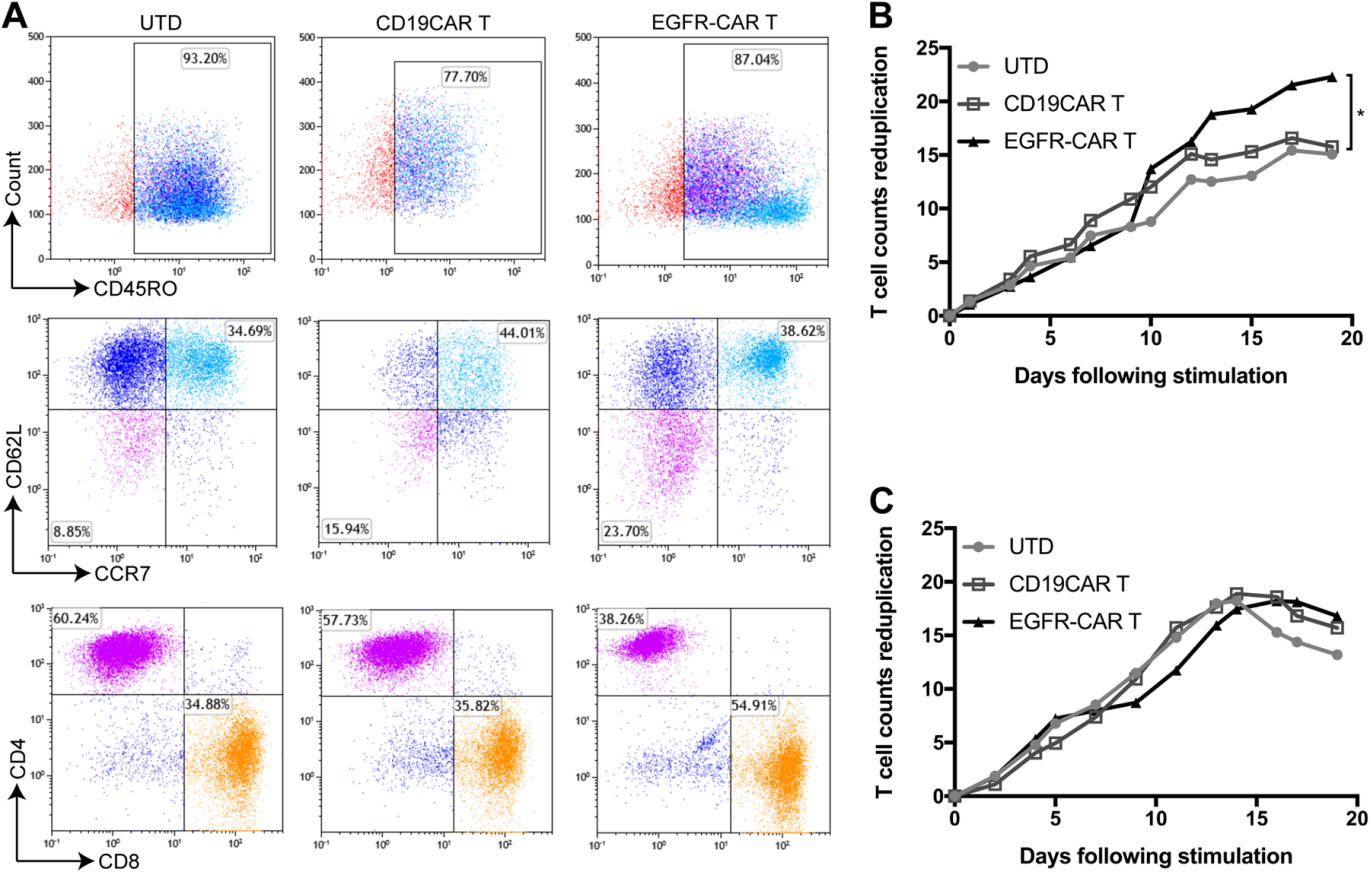
Characterization of transfected and activated CAR T cells. **a** Flow cytometry. Phenotypic and subset composition of CAR T-cell population was assayed by flow cytometry after 24 h stimulation. **b** CCK8 assay. Proliferation of CAR T cells during expansion in response to repetitive stimulation with EGFR antigen. **c** Cell counting assay. Proliferation of CAR T cells stimulated with CD3/CD8-activating beads was assayed using cell-counting assay. *P < 0.05

We then assessed these T cells using different functional assays and found that >38.6% of the CAR-T-EGFR cell population comprised central memory cells (CD45RO+CCR7+CD62L+), whereas 23.7% of these cells were effector memory cells (CD45RO+CCR7−CD62L−) (Fig. 2a). After co-culture with H23 cells for 48 h, the expression of CD3, CD4, and CD8 was evaluated in these T lymphocytes.

Our data showed that there was a higher proportion of CD3^+^CD8^+^ EGFR-CAR T cells (54.91%) compared with UTD cells (34.88%), indicating that the cytotoxic T lymphocyte (CTL) population increased significantly after co-culture of these EGFR-CAR T cells with tumor cells (Fig. 2a and Figure S1). Moreover, in addition to routine activation with coated CD3/CD28 antibody, stimulation with EGFR antigen also allowed EGFR-CAR T cells to continually proliferate, whereas absence of antigen stimulation showed cell expansion arrest (Fig. 2b, c).

### In vitro specific and potent cytotoxicity of EGFR-CAR T cells against NSCLC cells

According to the literature, >60% of NSCLC expressed a high level of EGFR^4^. Therefore, NSCLC particularly benefitted from the EGFR-specific CAR T-cell therapy regimen. We first detected EGFR expression in a panel of eight human NSCLC cell lines (Figure S2A). Flow cytometric analysis validated a high level of EGFR expression on the surface of lung cancer cell lines A549, HCC827, Calu-6, H23, H460, H292, and H1975 but a low level on the H1299 cell surface.

**Fig. 3:**
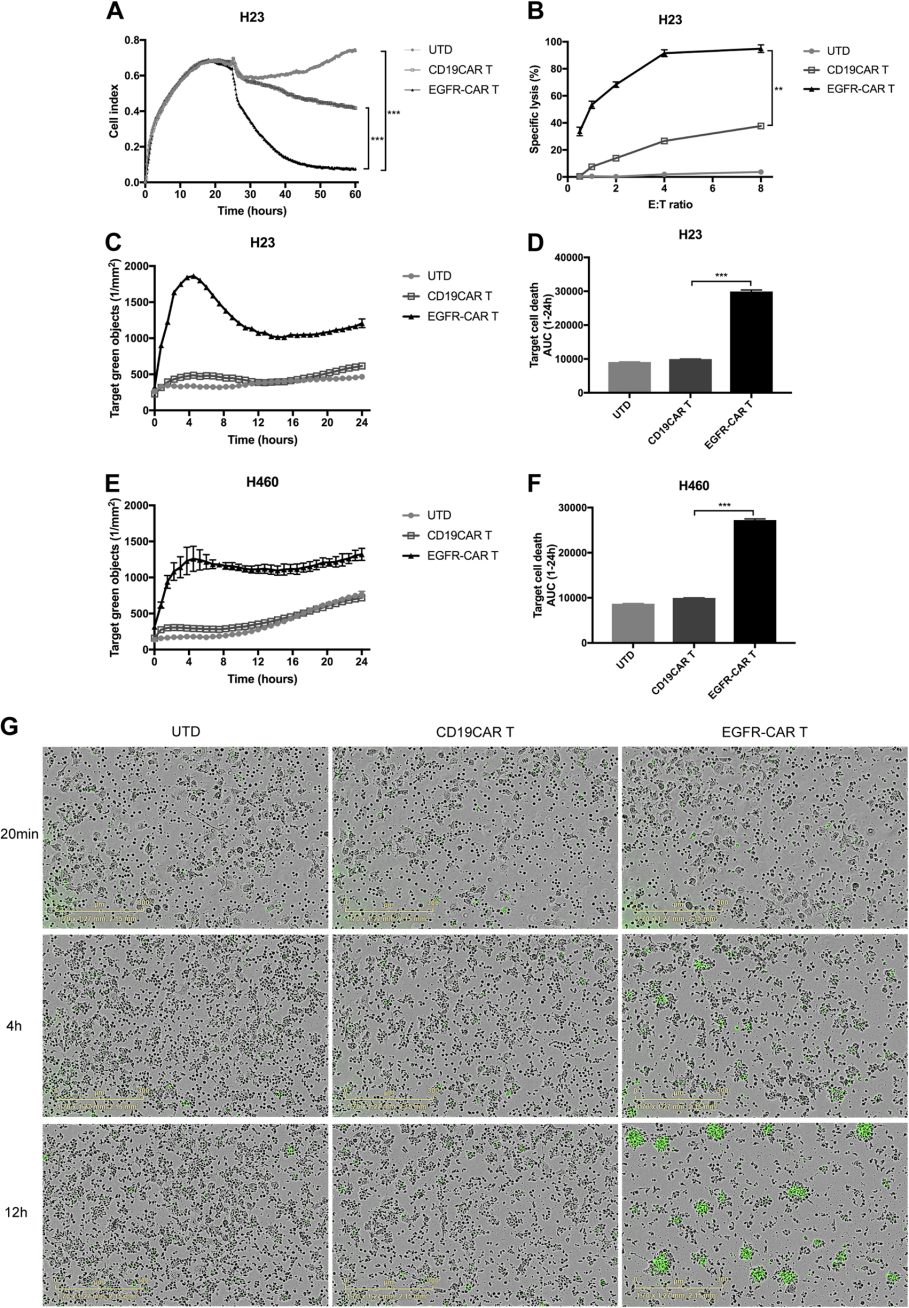
Cytotoxic activities of EGFR-CAR T lymphocytes against lung cancer cells in an EGFR-dependent manner in vitro. **a** Cytotoxicity of EGFR-CAR T cells against H23 cells detected by using the RTCA system at the E:T ratio of 8:1. **b** Quantified data on the specific lytic levels of CAR T cells against H23 cells extracted from RTCA data at different E:T ratios. **c**, **e** EGFR-CAR T-cell killing of H23 and H460 cells, respectively, were observed by using IncuCyte zoom. H23 and H460 were incubated with CAR T cells at an E:T ratio of 8:1. **d**, **f** Quantified data of **c**,** e**. **P < 0.01 and ***P < 0.001. **g** The sequential images of immune-cell killing of lung cancer cells labeled with YOYO-1, a green dead cell-labeling reagent. Representative merged images are shown for H23

We then performed different cell killing assays and cytokine productions to validate the specific lytic function of EGFR-CAR T cells against NSCLC cells in vitro. In particular, T cells precisely and efficiently expressed EGFR-CAR lysed EGFR^+^ NSCLC H23 and H460 cells but not EGFR-negative gastric cancer HGC27 cell line (Fig. 3a and Figure S2B, C), indicating that these re-engineered T cells acquired specific cytolytic activity against EGFR^+^ tumor cells. Meanwhile, we also found that these EGFR-CAR T cells exhibited increased cytotoxic activity at an appropriate ratio of E:T but not at lower ratios (Fig. 3b). Our real-time quantitative cell-killing assay using IncuCyte-FLR technology also showed similar cytotoxic data (Fig. 3c–g). The time course of H23 cell killing further revealed that tumor cells death occurred mainly after 4 h of EGFR-CAR T-cell co-culture and most cells were killed within 12 h of co-incubation.

**Fig. 4:**
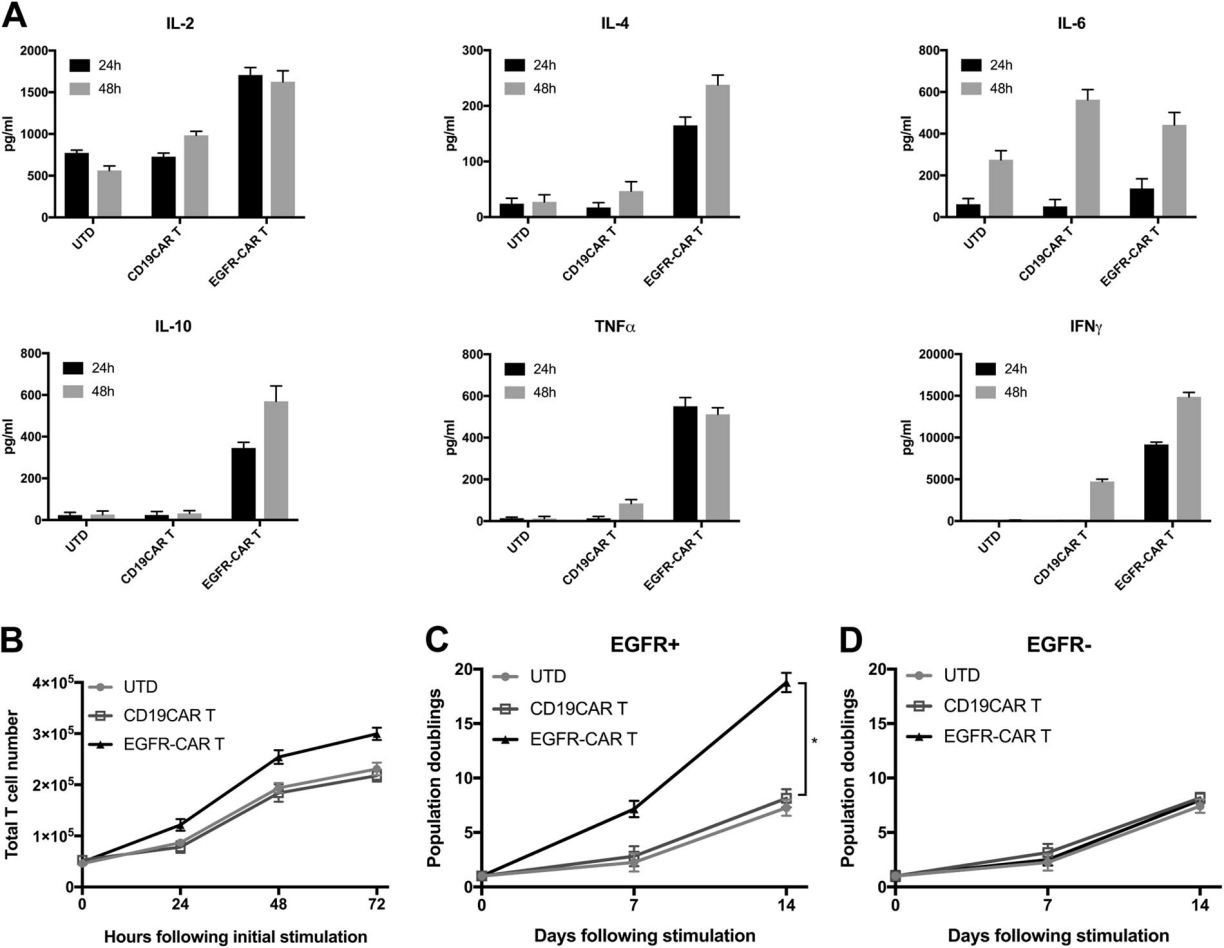
The in vitro activity of EGFR-CAR T cells in NSCLC cells. **a** Results of cytokine release assay. The level of different cytokines, including IL-2, IL-4, IL-6, IL-10, TNF-α, and IFN-γ, was assayed in the supernatants of co-culture of the effector cells with the target cells at an E:T ratio of 1:1 for 24 and 48 h. The effector cells were EGFR-CAR, UTD, and CD19CAR T cells. The target cells were human lung carcinoma H23 cell line. **b** Cell viability CCK8 assay. The total T-cell numbers were increased after co-culture with EGFR-expressing lung carcinoma cells at a ratio of 2:1. **c**, **d** Cell proliferation assay. The CAR T-cell expansion was measured in response to stimulation with EGFR-positive or -negative target cells at a 2:1 ratio. *P < 0.05. Curves reflect means ± SD for triplicate cultures of three independent experiments

In accordance with the cytotoxicity data, we also found high levels of cytokines (IL-2, IL-4, IL-10, tumor necrosis factor (TNF-α), and interferon (IFN)-γ) were released in H23 cells after 24 h co-incubation with EGFR-CAR T cells in an EGFR-specific manner (Fig. 4a). EGFR-CAR T cells progressively produced considerable higher levels of Th-2 cytokines, like IL-4, IL-6, and IL-10, after 48 h co-culture. As expected, UTD and CD19 CAR T cells only produced and released modest levels of cytokines after co-culture with EGFR-positive tumor cells, compared with EGFR-CAR T.

To determine the impact of antigen-stimulation on proliferation of redirected T cells, we performed both Cell Counting Kit-8 (CCK8) and cell counting assays and found that a significant augmentation in total EGFR-CAR T-cell population occurred after EGFR stimulation (Fig. 4b). Moreover, the cell counting assay showed that [in response to specific antigen activation] EGFR-CAR T cells enlarged and expanded after 2 weeks, in contrast to the absence of EGFR, thus demonstrating a proliferation advantage of cells after EGFR co-stimulation (Fig. 4c, d). Hence, human T cells transduced with EGFR-directed CAR showed a specific lytic activity, cytokine secretion, and proliferation in response to EGFR in vitro.

### Efficacy of EGFR-CAR T cells on NSCLC cell mouse xenografts

**Fig. 5:**
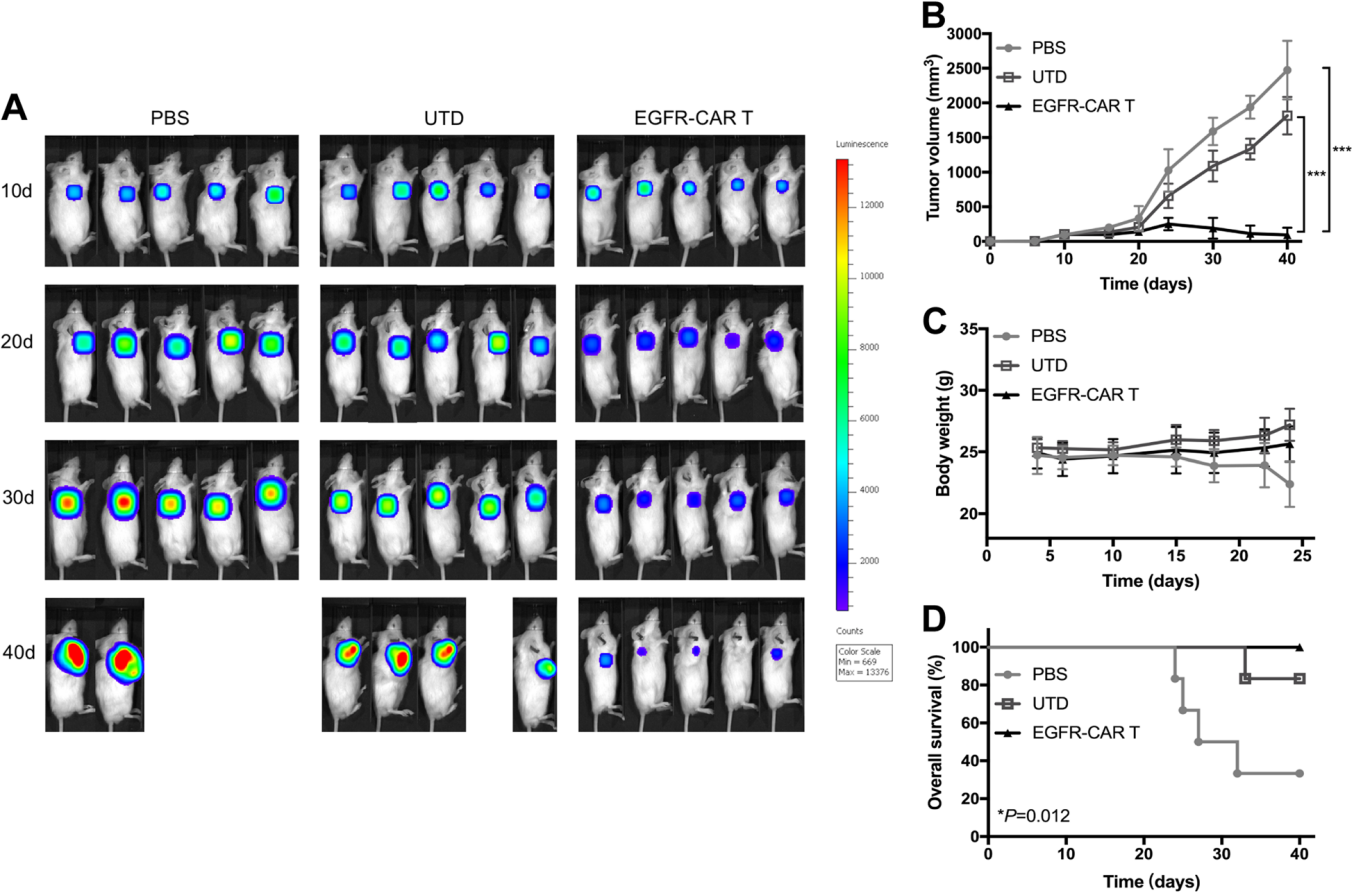
Effects of human EGFR-CAR T cells on reduction of NSCLC cell xenografts in mice. **a** NSG mice were injected with 5.0 × 10^6^ H460-fLuc cells for xenograft formation and growth in mice and then injected with 1.0 × 10^7^ CAR or un-transduced T cells intratumorally twice on days 0 and 7. Bioluminescence imaging was used to measure tumor growth. **b** Tumor xenografts in mice treated with EGFR-CAR T cells regressed compared to the other two groups. ***P < 0.001. **c** Mouse body weight. **d** Kaplan–Meier survival analysis of efficacy of CAR T cells in vivo. The survival curve was analyzed by the log-rank test

Next, we characterized the effect of these EGFR-CAR T cells on NSCLC cell mouse xenografts. Non-obese diabetic/severe combined immunodeficient/γ-chain^−/−^ (NSG) mice were first subcutaneously injected with 5.0 × 10^6^ H460-firefly luciferase (fLuc) cells, and when tumor volume reached approximately 200 mm^3^, 1.0 × 10^7^ CAR T and UTD T cells (or phosphate-buffered saline (PBS) for the same volume) were subcutaneously injected into tumor mass, and tumor xenografts were monitored via caliper-based sizing, bioluminescence imaging (BLI), and body weight for 40 days (Fig. 5a). Our data showed that EGFR-CAR T cells dramatically decreased tumor burden compared with those of PBS or UTD T cells (*P *< 0.001; Fig. 5b). Intratumoral delivery of EGFR-specific CAR T-cell therapy was associated with a notably better survival (Fig. 5d), but there was no weight loss observed in NSG mice treated with CAR T cells compared with PBS (Fig. 5c). In contrast, mice treated with PBS or UTD T cells reached a humane experimental end point by 42 days after initial tumor modeling.

To assess the potential toxicity of EGFR-CAR T cells, susceptible murine organs, including the lung, heart, liver, spleen, and kidney, were excised and examined histologically. No off-target toxicity was detected against these indicated organs after injection of EGFR-CAR T cells (Figure S3A). Similarly, there was no significant infiltration of CD3 T cells observed immunohistochemically (Figure S3B). In the spleen specimens, the engraftment and persistence of EGFR-CAR T cells were shown after immunohistochemical staining of CD3 (Figure S3C). In contrast, CD3-positive T cells were undetectable in the spleen specimens of UTD or PBS mice. Signs of graft-vs.-host disease and other toxicity (such as changes in the mouse body weight, loss of fur, conjunctivitis, and diarrhea) were not observed.

## Discussion

In the current study, we mimicked and modified an immunotherapeutic strategy against hematological malignancies using EGFR-CAR T-cell therapy of NSCLC in vitro and in nude mouse xenografts. Our data showed: (i) the *piggyBac* transposon-based gene transduction was effective; (ii) these EGFR-CAR T cells possessed a higher proportion of CD3^+^CD8^+^ CTL population with the proliferative ability; and (iii) in vitro- and in vivo-specific and potent cytotoxicity against NSCLC cells. Considerable concern surrounds the efficacy of scFv-based CAR T cells that target CD19 and other specific antigens in the treatment of advanced B-cell malignancies^16–20^, although the US Food and Drug Administration recommended approval of CTL019 (the first CAR T-cell therapy) for treatment of relapsed or refractory patients with B-cell ALL^21^. A recent study of solid tumors using this approach also gave positive results^22^ and two clinical trials demonstrated that the re-engineered CAR T cells could lead to durable objective regression in different malignancies including neuroblastoma, sarcomas, and metastatic melanoma^23,24^. In our current study, we generated an EGFR-CAR using *piggyBac* transposon-based gene transduction and revealed its therapeutic potential against EGFR-positive human cancers, such as NSCLC.

The CAR T cells engineered in our research laboratory possessed the EGFR antigen-dependent expansion and antitumor activities. First, we found that a multitude of NSCLC cell lines expressed a high level of EGFR, while co-culture with these EGFR-CAR T cells displayed T-cell proliferation capacity, T-cell phenotype, and cytokine secretion in an antigen-specific manner since the UTD T cells were at growth arrest and did not produce and secrete various cytokines. We also showed that these T cells were the effector and memory phenotype T cells because of high levels of T helper type 1 (Th1) cytokines generated with EGFR-positive tumor cells, thus indicating the cytotoxic effect of these EGFR-CAR T cells. Interestingly, the exception associated with these EGFR-CAR T cells was the greater IL-4 and IL-10 production, suggesting that intracellular combination immunoreceptor tyrosine-based activation motif activation enforced a Th1 distortive response. In addition, we also assessed these EGFR-CAR T-cell antitumor activities using two different cytotoxic assays.

The *piggyBac* transposon system is an alternative approach for the safe and effective gene transduction for CAR T lymphocyte therapy^17^. Previously, integrated viral vectors, such as retroviral and lentiviral protocols, have been used to introduce the CAR transgene as the most popular strategy for CAR T-cell clinical applications^25^. However, several concerns are associated with the clinical application of virus-mediated gene transduction, including cost of virus manufacture, complexity of production processes, random integration into the genome of lymphocytes, and unexpected immune-mediated toxicity caused by long-term persistence in cells^14,17^. In contrast, the *piggyBac* transposon system showed a more stable transduction efficiency, potentially a good manufacturing practice compliant system, and a larger production capacity of transgenes and dispensation with CAR T cells' enrichment^26,27^. In our current study, 30–45% of the transfection efficiency into peripheral blood mononuclear cells (PBMCs) was reached, and antitumor activity lasted 3 weeks after optimizing the electric transfer parameters and culture conditions. Thus this non-viral transfection system was sufficiently efficacious, and in the future it could replace viral CARs in vitro and in clinical studies.

Furthermore, our current study also showed that EGFR-specific CAR T-cell therapy was safe and had no off-target effects on mice (the usual concern in immunotherapy research)^28^. EGFR is expressed on the cell surface of a wide variety of epithelium-derived solid tumors and most normal epithelial tissues, and the off-tumor toxicity related to CAR T-cell therapy is concerning^12^. Based on cetuximab’s clinical toxicological data, the most likely toxicities are skin rash and diarrhea^29^. Higher affinity of scFv to EGFR is required for efficient combination to discriminate the damage activities between EGFR-overexpressed cancer cells and relatively low EGFR-expressed normal cells^30–32^. For example, a phase I clinical study using the secondary generation lentivirus-transduced EGFR-CAR T cells had tolerable and controllable target-related toxicities in 11 NSCLC patients^33^. In our current study, we histopathologically examined the potential systemic toxicity in different murine organs and found that this EGFR-CAR T therapy did not cause notable pathological changes and exogenous T lymphocyte infiltration in major murine organs.

To enhance CAR T-cell persistence and anticancer efficacy, many research teams have exploited potential therapeutic strategies to overcome the hurdles for CAR T-cell therapy in solid tumors^34^. The combination of adoptive T-cell regimen and other immunotherapies is a potentially promising strategy to overcome the unsatisfactory curative effect for solid tumors and the relapse after immune checkpoint inhibitor treatment. Chong et al. (2017), in a clinical study of refractory and progressive lymphoma, has proven that the administration of the programmed death receptor-1 (PD-1) blocking antibody after adoptively CAR T cell regimen can reach a clinically significant antitumor activity^35^. Collectively, patients with solid malignancies will benefit from combined immune checkpoint blockade of co-inhibitory pathways, including PD-1, cytotoxic T-lymphocyte-associated protein 4, V-domain Ig suppressor of T cell activation, and lymphocyte activation gene 3 (LAG-3)^36^. The design of T cells modified with both CARs engineered with one or two checkpoint inhibitors could allow maximum T-cell activation in tumor lesions and effective reduction of a systemic toxicity^37^. Ultimately, autologous T lymphocyte re-engineering provides opportunities to optimize combination strategies to improve CAR construction that may maximize CAR T-cell therapy against solid tumors.

Some limitations in our current study need to be acknowledged. For example, because of the lack of competent immune system, NSG mice could not simulate the reaction to human antigens, like EGFR, leading to the cytokine release syndrome^38^; thus the true on- or off-target effects need further investigation. Additionally, a more readily available relevant human “normal tissue” model is needed for safety evaluation.

In conclusion, our current study on the non-viral *piggyBac* transposon system for treatment of EGFR-positive NSCLC demonstrates it to be an effective, inexpensive, and safe protocol to generate the EGFR-specific CAR T cells. The EGFR-CAR T therapy may also be a potential strategy for other solid malignancies with EGFR overexpression. Based upon our current data, we have designed the immune checkpoint inhibitor expressing EGFR-CAR T regimen to overcome the concerns caused by tumor microenvironment, which leads us to an imminent phase I clinical trial (NCT03030001).

## Materials and methods

### Cell lines and culture

Human NSCLC A549, H23, and H1299 cell lines were purchased from American Type Culture Collection (Manassas, VA, USA), while H460, H292, HCC827, H1975, HGC27, and Calu-6 cell lines were obtained from Chinese Academy of Sciences (Shanghai, China). These cell lines were maintained in Roswell Park Memorial Institute medium-1640 (RPMI-1640) or RPMI-1640 mixed with Dulbecco’s modified Eagle’s medium (Gibco BRL, Gaithersburg, MD, USA) supplemented with 10% heat-inactivated fetal bovine serum (Gibco) in a humidified incubator with 5% CO_2_ at 37 °C. H460 cells were also transduced with the firefly luciferase (fLuc) through lentiviral transduction and puromycin selection.

### Generation of the CAR-modified human T lymphocytes

The EGFR-BBz CAR encoding the EGFR scFv with 4-1BB co-stimulatory and CD3ζ endodomains was PCR-amplified and cloned into the *piggyBac* transposon vector pNB328-EF1α to obtain the recombinant plasmid pNB328-EGFRCAR. The pNB328-CD19CAR and blank pNB328 were used as negative controls. After amplification, these plasmids were confirmed by DNA sequencing.

PBMCs were isolated using the Ficoll density gradient centrifugation methods from whole blood of healthy volunteer donors and obtained from The Department of Blood Transfusion, The Second Military Medical University (Shanghai, China). This study was approved by the Ethics Committee of The Second Military Medical University and each donor signed an informed consent document beforehand.

For our experiments, PBMCs were placed in an incubator for 2–4 h, and 5.0 × 10^6^ floating cells were collected and resuspended in the buffer out of the Human T Cell Nucleofactor Kit (Lonza, Switzerland) and added separately with the *piggyBac* transposase and recombinant transposon plasmids using electroporation according to the manufacturer’s instructions. PBMCs were cultured and stimulated with the anti-CD3/CD28 beads (Invitrogen, Carlsbad, CA, USA) in the AIM V medium (Gibco) supplemented with IL-2 for 10–14 days at 37 °C in a humidified incubator with 5% CO_2_. The T-cell expansion was monitored through the total cell counts following three rounds of stimulation with specific antigen or T-cell activation antibodies to obtain CAR T cells.

### Western blot

CAR T cells were lysed and quantified using a standard protocol according to a previous study^39^. Protein samples were separated in 10% sodium dodecyl sulfate polyacrylamide gel electrophoresis gel and transferred onto polyvinylidene fluoride membranes (Roche, Basel, Switzerland). For western blotting, the membranes were blocked in 5% non-fat powdered milk solution in PBS for 1 h at room temperature and then blotted with different primary antibodies of mouse anti-GAPDH (anti-glyceraldehyde 3-phosphate dehydrogenase; Beyotime, Shanghai, China) and mouse anti-CD3ζ (Abcam, Cambridge, UK) at the manufacturers’ recommended dilutions at 4 °C overnight. The following day, levels of immunoreactive protein bands were visualized using the enhanced chemiluminescence (BioVision, San Francisco, CA, USA), after being washed with PBS-Tween 20 and incubation with a horseradish peroxidase-conjugated goat anti-mouse IgG (H+L) secondary antibody (Beyotime), and then scanned and quantified using the Image Lab™ Software (Bio-Rad, Hercules, CA, USA). GAPDH was used as an internal control.

### Quantitative reverse transcriptase-PCR

Total RNA was isolated from of CAR T cells using a TRIzol reagent (Invitrogen) and reversely transcribed into cDNA using the Quantscript RT Kit (Tiangen Biotech Co., Ltd., Beijing, China) according to the manufacturers’ protocols. The primer recognizing the 4-1BB-TCRζ junctional fragment was set to detect transgene nucleic acid. Quantitative PCR was then performed using the SYBR Green qPCR Master Mix (Thermo-Fisher, Waltham, MA, USA) and primers to amplify *CAR* and *β-Actin* cDNA were: *CAR*, 5′-CTCCTGCACAGTGACTACATG-3′ and 5′-GAACTTCACTCTGGAGCGATAG-3′; *β-Actin*, 5′-CTCCATCCTGGCCTCGCTGT-3′ and 5′-GCTGTCACCTTCACCGTTCC-3′. The quantitative PCR conditions were set to 95 °C for 5 min and then 40 cycles of 95 °C for 15 s, 60 °C for 30 s, and 72 °C for 30 s. The relative mRNA level was normalized to endogenous control β-actin and calculated based on the 2^−ΔΔCt^ comparative method. All reactions were performed in triplicate and repeated at least once.

### Flow cytometry

Expression of EGFR and other proteins on the tumor cell surface was detected by flow cytometry using the anti-EGFR antibody (BD, San Jose, CA, USA). In brief, the CAR T cells were collected from culture and assayed with monoclonal antibodies against human CD25-PC5 and CD69-PC5 (Beckman Coulter Inc., Indianapolis, IN, USA), CD3-PE-CY5, CD4-PE, CD8-FITC, CD45RO-PE-CY5, CD62L-PE, TIM3-PE, and LAG3-Alexa Fluor 647 (Biolegend, San Diego, CA, USA) and CCR7-FITC, CD107α-PE-CY5, and PD-1-PE (BD, San Jose, CA, USA) according to a previous study^40^ or the manufacturers’ instructions.

EGFR-modified CAR expression was detected by using an indirect method with biotinylated EGFR protein and streptavidin-coupled PE antibody (BD). Fluorescence was assessed using a Beckman Coulter Gallios™ flow cytometer, and the data were analyzed with the FlowJo vX.0.7 and Kaluza v1.5 software (Beckman Coulter Inc.).

### Cell viability and proliferation assay

Short-term proliferation of CAR T cells co-cultured with H23 cell line was assessed using the CCK8 (MedChemExpress, Monmouth Junction, NJ, USA). Specifically, H23 cells were seeded into 6-well plates in triplicates at a density of 5.0 × 10^5^ cells per well and cultured for 24 h and the CAT T cells were then added into the cell culture at a ratio of 1:1 in the absence of IL-2 and co-cultured for up to 3 days. At the end of the experiments, CCK8 solution was added into each well and the cells were further incubated at 37 °C for 1 h. The optical density was measured at 450 nm with a reference wavelength of 630 nm using a spectrophotometer (BioTek Instruments, Winooski, VT, USA).

The numbers of T cells were counted using the Countess II FL Automated Cell Counter (Invitrogen) after Trypan blue exclusion.

### Flow cytometric cytokine release assay

CAR-transduced and UTD T cells were co-cultured with cancer cells at a ratio of 1:1 in 6-well plates at a density of 5.0 × 10^5^ cancer cells per well in a volume of 3 ml medium. After 24 and 48 h culture, the cell culture medium was collected and measured for secretion of IL-2, IL-4, IL-6, IL-10, TNF-α, and interferon γ (IFN-γ) using flow cytometry with Cytometric Bead Array (CBA; BD Biosciences). The data were expressed as the mean of triplicate wells ± SE.

### Cytotoxicity assay

We utilized two different cytotoxic function assays based on CELLigence real-time cell analyzer (RTCA) System (ACEA Biosciences, San Diego, CA, USA) and IncuCyte-FLR-Platform (Essen BioScience, Ann Arbor, MI, USA), respectively. The impedance-based RTCA was used for label-free and real-time monitoring of cytolysis activity. The cancer cells were seeded at a density of 1.0 × 10^4^ cells per well and grown for 24 h. The CAR and control T cells were added into the RTCA unit at different effector/target (E:T) ratios. The impedance signals were recorded by using the instrument for duration of 72 h in 5 min intervals.

Cytotoxicity of the CAR-expressing T cells was also tested by using the IncuCyte zoom. Tumor cells were plated into a 96-well plate at a density of 5.0 × 10^4^ cells per well in triplicate. After cells adhered onto the plates, T cells were added into each well at a final volume of 150 μl per well with dissimilar E:T ratios. Images were taken every 30 min and the number of dead cells was quantified using the YOYO-1 (Thermo-Fisher), which is a green fluorescent dye to stain cell DNA. The cell killing activity was analyzed using the IncuCyte Zoom live cell imaging system. The assays were repeated three times, and the data were summarized as mean ± SD.

### Animal experiments

The animal protocol of this study was approved by the Institutional Animal Care and Use Committee (IACUC) of the Second Military Medical University, Shanghai. Female NSG mice aged 6–8-week were purchased from Beijing Vitalstar Biotechnology Co. Ltd (Beijing, China) and maintained in a specific pathogen-free “barrier” facility and housed under controlled temperature and humidity and alternating 12 h light and dark cycles. The NSG mice lack mature T cells, B cells, and natural killer cells^41^; thus, they are better than nu/nu mice for our study of EGFR-CAR T cells. For our experiments, the mice were inoculated subcutaneously with 5.0 × 10^6^ H460-fLuc cells in 100 μl of PBS on the flank. After the tumor became palpable, 18 mice were chosen with similar tumor sizes and randomized into three groups, including PBS, UTD, and EGFR-CAR T groups, to lead the tumor burden to reaching to approximately 200 mm^3^. The mice were treated with 1.0 × 10^7^ CAR T cells intratumorally; this was repeated 1 week later and the control mice were treated with PBS or UTD T cells. Tumor progression was confirmed by BLI using a Xenogen IVIS imaging system (PerkinElmer, Hopkinton, MA, USA) for up to 40 days. Tumor volume was calculated with the formula: *V* = ½ (length × width^2^).

### Statistical analysis

The data were expressed as means ± standard deviation and analyzed using Student’s *t*-test, while the statistical comparison between groups was performed using two-way repeated-measures analysis of variance for the tumor burden. Survival curve was analyzed using the Kaplan–Meier curves and log-rank test. *P*-values < 0.05 were considered statistically significant. All statistical analyses were performed by using GraphPad Prism v7.0 (GraphPad Software, La Jolla, CA, USA).

## Electronic supplementary material

Supplementary materials
