# Supplementary material for: Antitumor activity of EGFR-specific CAR T cells against non-small-cell lung cancer cells in vitro and in mice
Source: Cell Death Dis. 2018 Feb 7;9(2):177. doi: 10.1038/s41419-017-0238-6 (PMC5833445; doi:10.1038/s41419-017-0238-6)
Supplement: Supplementary file 1 — Supplementary materials [file 41419_2017_238_MOESM1_ESM.doc]

**Antitumor activity of EGFR-specific CAR-T cells** **against non-small-cell lung cancer cells in vitro and in mice**

**Supplementary Figure Legends**

**Figure S1. Flow cytometric analysis of the activation and exhaustion markers in CAR T cells.** The expression of CD25, CD69, PD-1, TIM-3, and LAG-3 in CAR T lymphocyte population was assayed by flow cytometry after 24 h stimulation.

**Figure S2. Expression of EGFR protein in different NSCLC cell lines. A,** Flow cytometry. Levels of surface EGFR expression (solid black line) or isotype antibody control (filled gray histogram) in various human lung carcinoma cell lines were detected by FCM. **B****,** Cytotoxicity assay. Specific cytotoxic activity of EGFR-CAR T cells against EGFR^+^ H460 cells. **C,** Cytotoxicity assay. Cytotoxic activity of EGFR-CAR T cells against EGFR^-^ HGC27 cells. ****P*<0.001.

**Figure S3. Safety evaluation after CAR T therapy *in vivo*. A** and **B,** H&E and immunohistochemistry, respectively. The data show that there is no off-target toxicity against mouse heart, lung, liver, and kidney. ×100 magnifications. Scale bars, 150 μm. **C,** H&E. EGFR-CAR T cell persistence and localization in the spleen *in vivo* is mildly antigen-specific. ×400 magnifications. Scale bars, 75 μm.


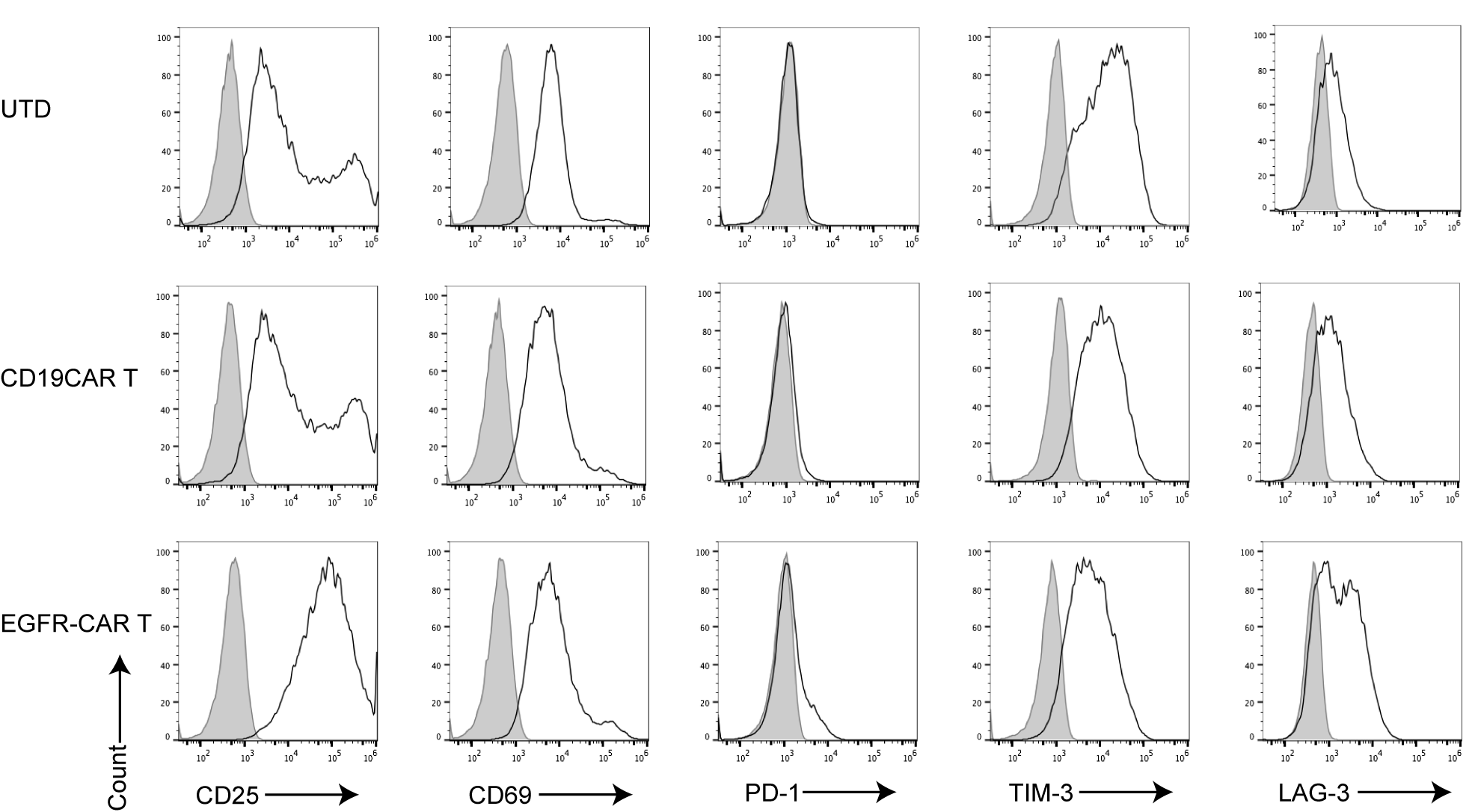


**Figure S1**

**
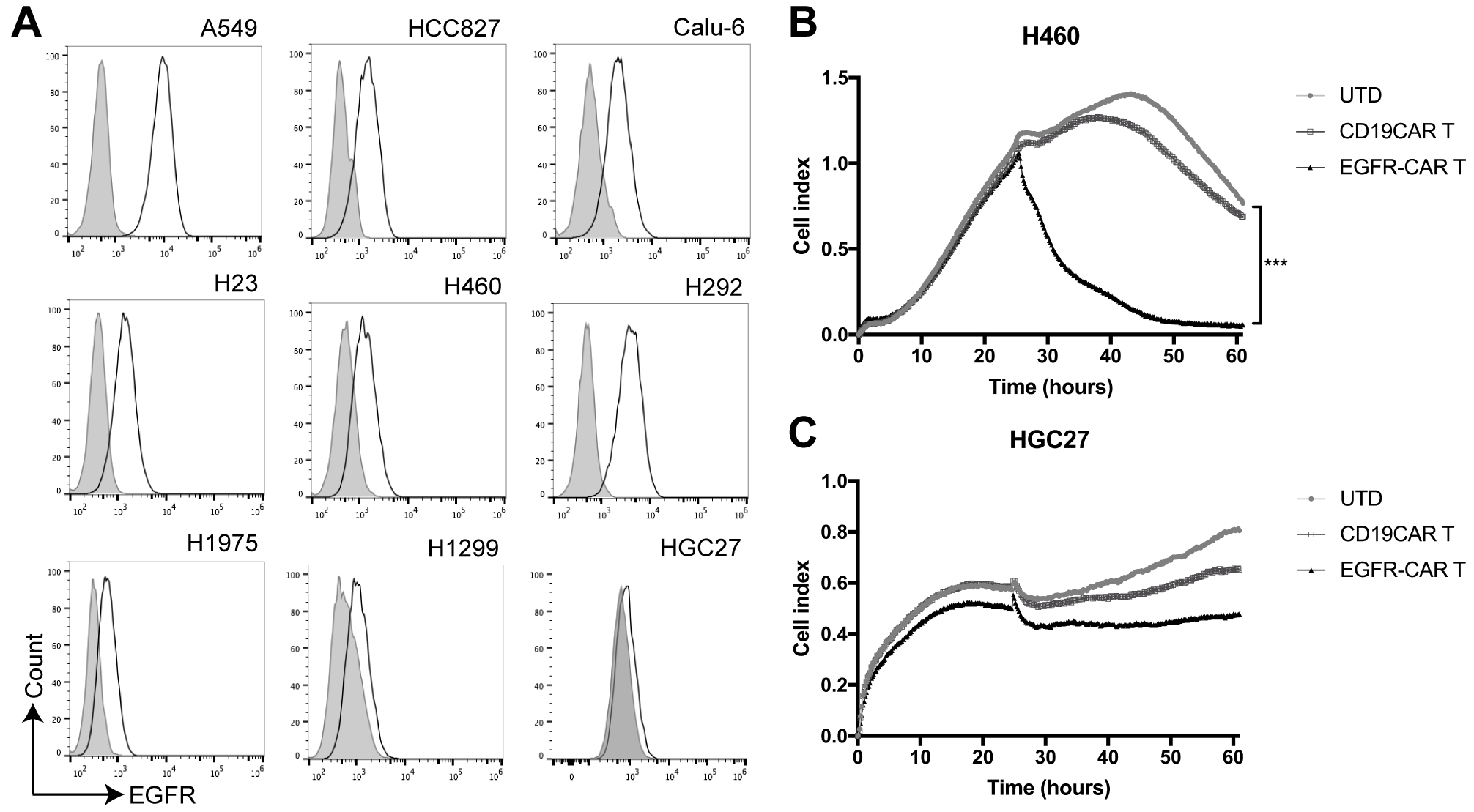
**

**Figure S2**

**
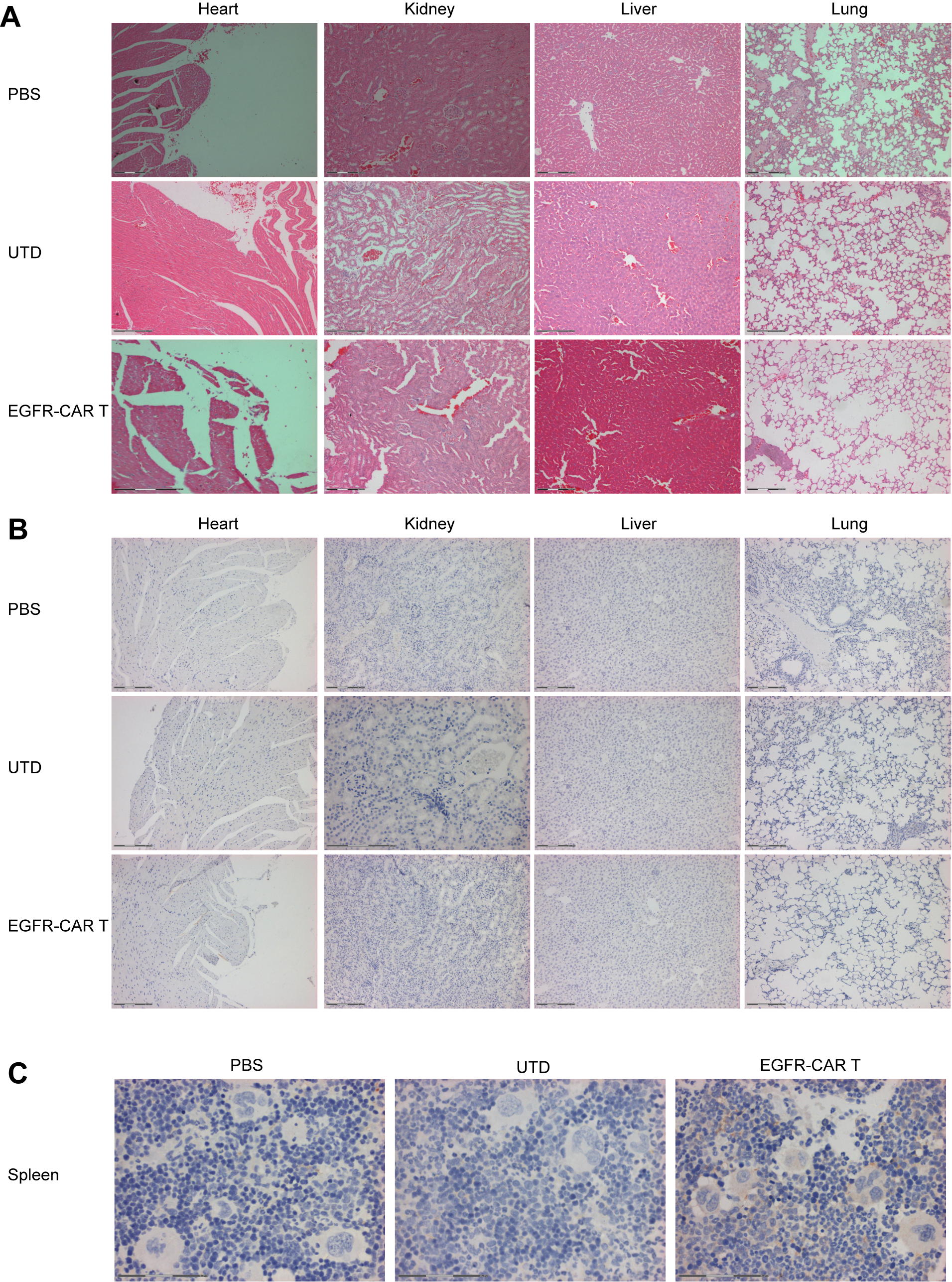
**

**Figure S3**
